# Isolation and Molecular Characterization of Three *Staphylococcus pseudintermedius* Strains from Dogs and Humans in Egypt

**DOI:** 10.1007/s00284-025-04477-7

**Published:** 2025-09-06

**Authors:** Haitham Elaadli, Yassien Badr, May Raouf, Stephen A. Kania, Ola K. Elsakhawy, Hend Altaib, Mohamed A. Abouelkhair

**Affiliations:** 1 Department of Animal Hygiene and Zoonoses, Faculty of Veterinary Medicine, Alexandria University, Alexandria, 22758 Egypt; 2 Department of Infectious Diseases and Epidemics, Faculty of Veterinary Medicine, Damanhour University, Damanhour, 22511 Egypt; 3 Department of Medical Microbiology and Immunology, Faculty of Medicine, Alexandria University, Alexandria, 21131 Egypt; 4 Department of Biomedical and Diagnostic Sciences, University of Tennessee, Knoxville, TN USA; 5 Department of Diagnostic Medicine and Pathobiology, Rowan University, Glassboro, NJ USA; 6 Research and Development Department, Middle East for Vaccines (MEVAC), El Sharqia, 44813 Egypt

## Abstract

*Staphylococcus pseudintermedius* is an opportunistic pathogen that is largely associated with canine hosts but is becoming more widely recognized as a zoonotic pathogen. Understanding its genetic and phenotypic properties, such as virulence factors and antimicrobial resistance (AMR) profiles, is critical for infection control and vaccine development. In this study, we isolated and molecularly characterized three *S. pseudintermedius* isolates from dogs (hereafter referred to as *S. pseudintermedius* D8) and humans (hereafter referred to as *S. pseudintermedius* H10 and *S. pseudintermedius* H11) in Egypt. All three isolates showed 100% sequence identity with the *nuc* gene of the *S. pseudintermedius* SP_11304-3A reference genome. Multilocus sequence typing (MLST) revealed novel sequence types (STs) in the three isolates. The AMR determinants varied substantially among the isolates. While the *mecA* gene was absent, *blaZ* was detected in the canine isolate, indicating beta-lactamase-mediated penicillin resistance. Additionally, *tetK* and *tetM* genes were found conferring tetracycline resistance in different isolates. Resistance genes for aminoglycosides, chloramphenicol, fusidic acid, macrolides, streptothricin, and trimethoprim were also identified. All isolates were positive for key virulence genes, including immune evasion (*AdsA*), coagulase (*coa*), immunoglobulin-binding protein (*sbi/spsK*), exfoliative toxin (*speta*), enterotoxins (*se-int* and *siet*), fibrinogen binding protein gene (*fnbB*), and two-component pore-forming leukocidin genes (*lukF* and* lukS*). The *S. pseudintermedius* H11 isolate uniquely harbored the neuraminidase gene (*nanB*), while none of the isolates contained the gene coding for immunoglobulin G binding protein (*spsQ*). These findings highlight the differences in virulence and antimicrobial resistance genes among these *S. pseudintermedius* isolates, underlining the need for global surveillance and molecular characterization of this pathogen.

## Introduction

Species of the *Staphylococcus* genus are commensal bacteria residing on the skin surface and upper respiratory tract mucosa of animals and humans; however, they can result in opportunistic infections in both hosts [1]. They are usually divided into two categories, based on coagulase reactivity, coagulase-positive staphylococci (CoPS) and coagulase-negative staphylococci (CoNS) [2]. Among CoPS, *Staphylococcus intermedius* group (SIG) is comprised of five distinct staphylococcal species, including *Staphylococcus intermedius*, *Staphylococcus pseudintermedius*, *Staphylococcus cornubiensis*, *Staphylococcus delphini*, and *Staphylococcus ursi* [3].

*Staphylococcus pseudintermedius* (formerly classified with *S. intermedius*) is a Gram-positive, coagulase-positive opportunistic bacterial pathogen that colonizes the skin and mucocutaneous sites including nose, mouth, and anus mostly in dogs and to lesser extent in cats [4]. Furthermore, It has particular veterinary significance, primarily in dogs, being frequently associated with dermatological afflictions such as canine pyoderma, abscesses, otitis externa, atopic dermatitis, and surgical wound infections [5].

*Staphylococcus pseudintermedius* is regarded as a newly evolved bacterial zoonotic pathogen and it has been increasingly reported throughout the world. It has been isolated from human infections including skin and soft tissue infections (SSTIs) [6–10], rhinosinusitis, pneumonia, otitis [7, 9, 11–13], bacteremia, endocarditis, and device-associated infections [7, 14, 15]. Moreover, it has been increasingly observed in people who come in close contact with dogs including dog owners and veterinary staff in pet clinics and veterinary teaching hospitals [7, 16–21]. In addition, it has been demonstrated that *S. pseudintermedius* can persist for a sustained period of time in the clinical environment and households where domestic dogs and their breeders live in close contact [22–24].

The correct species identification of* S. pseudintermedius* from animal and human samples is considered challenging because it can be misidentified as other CoPS, especially *Staphylococcus aureus* and *Staphylococcus intermedius,* when routine microbiological diagnostic methods are used. Thus, its occurrence as the causative infectious agent in human bacterial infections may have been underestimated [6, 9]. **P**olymerase **C**hain **R**eaction–**R**estriction **F**ragment **L**ength **P**olymorphism (PCR–RFLP) [25] and *nuc* PCR [26] are the primary molecular diagnostic techniques used to identify *S. pseudintermedius* at the species level. The former test relies on *Mbo*I restriction fragmentation of the amplified *pta* gene segment. The latter, makes use of species-specific oligonucleotides for amplifying a segment of the *nuc* gene (which encodes a thermostable endonuclease). Despite the increased total number of domiciled dogs and dog owners in Egypt, especially in urban regions, to the best of our knowledge, no study to date has investigated the detection of *S. pseudintermedius* either in dogs or humans in Egypt. The aims of this study were to molecularly characterize *S. pseudintermedius* isolated from dogs and humans in Egypt. Given the close contact between dogs and humans, the zoonotic potential of *S. pseudintermedius* poses significant public health concerns, particularly for pet owners and veterinary staff. Understanding the genetic and phenotypic properties of this pathogen helps to elucidate its virulence mechanisms, antimicrobial resistance, and provides important information regarding its molecular epidemiology. This information is crucial for developing effective infection control strategies and guiding therapeutic interventions.

## Materials and Methods

### Sample Collection

A cross-sectional study was performed between March 2022 and November 2022 in the Alexandria governorate, Egypt. A total of 174 pus swabs of septic wounds were collected from human patients in Alexandria University Hospital. In addition, a total of 67 apparently healthy and clinically diseased dogs having dermatological lesions such as pyoderma, wounds, or otitis externa were included in the study. The dogs were visiting different private pet clinics in the Alexandria governorate. The dog’s demographic and clinical data were obtained. A total of 124 samples, including skin, nasal, and mouth swabs, were taken from the 67 dogs (in some cases, several samples from different body sites were collected from the same individual dogs). All swabs were transported in tubes containing sterile nutrient broth and transferred on ice to the diagnostic laboratory of the Faculty of Veterinary Medicine, Alexandria University, for further bacteriological analysis.

### Bacterial Isolation and Identification

All specimens were individually inoculated by streaking on defibrinated 5% sheep blood agar and incubated aerobically overnight at 37 °C to identify beta hemolytic activity, following laboratory procedures at Faculty of Medicine, Alexandria University, Egypt. Catalase-positive, Gram-positive cocci were subcultured directly into mannitol salt agar for selective isolation of Staphylococci. Subsequently, the tube coagulase test, with rabbit plasma, was performed and only CoPS strains were selected and kept at −20 °C in brain heart infusion containing 20% glycerol for further molecular identification at the species level [27].

### DNA Extraction and Molecular Detection of *Staphylococcus pseudintermedius* by PCR Assay

DNA extraction was performed using the boiling method [28] after subculturing the CoPS isolates on nutrient agar plates. The extracted DNA was amplified using species-specific primers targeting the *nuc* gene of *S. pseudintermedius* [26]. The forward primer Sp-nuc-F 5′-TGATGCAGCTTTTCCGTATG-3′ and reverse primer Sp-nuc-R 5′-AAAGATGGGCAAGATGAACG-3′ produced a 99 bp amplicon as previously reported [29]. The PCR reaction mixture, with a total of 25 µl, consisted of 12.5 µl of Phusion™ Master Mix (Thermo Fisher Scientific, USA), 0.5 µl of each primer of 50 µM concentration (Integrated DNA Technologies, USA), 6.5 µl of nuclease-free water, and 5 µl of DNA template. The reaction mixture was transferred to a thermal cycler (Applied Biosystems, USA) and heated once at 95 °C for 2 min, followed by 40 cycles at 95 °C for 30 s, 60 °C for 30 s, and 72 °C for 1 min, and then a final extension at 72 °C for 5 min.

### DNA Sequencing

The *nuc* PCR products of human and dog isolates were purified using a QIAquick gel extraction kit (Qiagen, Valencia, CA). The purified products were sequenced in both directions using a BigDye Terminator v3.1 cycle sequencing kit (Applied Biosystems, Foster City, CA, USA) in an Applied Biosystems 3130 genetic analyzer (Applied Biosystems) according to the manufacturer’s instructions. BLAST v2.12.0 analysis (https://blast.ncbi.nlm.nih.gov/Blast.cgi?PROGRAM=blastn&PAGE_TYPE=BlastSearch&LINK_LOC=blasthome) was performed to confirm the nucleotide sequence identity.

### Multi-locus Sequence Typing

Multilocus sequence typing (MLST) was conducted using the 7-gene MLST scheme from the *S. pseudintermedius* MLST database (https://pubmlst.org/organisms/staphylococcus-pseudintermedius, accessed on July 18, 2024) [30]. The genes involved in the MLST analysis were *ack*, *cpn60*, *fdh*, *pta*, *purA*, *sar*, and *tuf*. The three isolates in this study were tested by conventional PCR amplification and subsequently sequenced as described earlier. The primers used for PCR are shown in Table 1.

**Table 1:** Primers for PCR amplification of the 7 loci for MLST typing of *S. pseudintermedius*

Locus	Primer type	Sequence (5′–3′)	Product size (bp)	References
*Tuf*	Forward	CAATGCCACAAACTCG	500	[31]
	Reverse	GCTTCAGCGTAGTCTA		
*cpn60*	Forward	GCGACTGTACTTGCACAAGCA	552	[31]
	Reverse	AACTGCAACCGCTGTAAATG		
*pta*	Forward	GTGCGTATCGTATTACCAGAAGG	570	[31]
	Reverse	GCAGAACCTTTTGTTGAGAAGC		
*purA*	Forward	GATTACTTCCAAGGTATGTTT	490	[32]
	Reverse	TCGATAGAGTTAATAGATAAGTC		
*fdh*	Forward	TGCGATAACAGGATGTGCTT	408	[32]
	Reverse	CTTCTCATGATTCACCGGC		
*ack*	Forward	CACCACTTCACAACCCAGCAAACT	680	[32]
	Reverse	AACCTTCTAATACACGCGCACGCA		
*sar*	Forward	GGATTTAGTCCAGTTCAAAATTT	521	[32]
	Reverse	GAACCATTCGCCCCATGAA		

The PCR consisted of 95 °C for 1.5 min, followed by 35 cycles of 30 s at 52 °C, 1 min at 72 °C, 30 s at 94 °C, and then 52 °C for 30 s, and a final extension at 72 °C for 5 min. PCR products were resolved and visualized by electrophoresis in gels containing 1.4% agarose and 1× SYBR Safe stain. PCR amplicons of expected sizes, produced from all isolates in the panel, were treated using enzymes to destroy single-stranded DNA (ExoSAP-IT; Thermo Fisher Scientific, USA), and purified samples were sent to a commercial laboratory (Eurofins) for Sanger sequencing. Sequences were de novo assembled using Geneious Prime®2024 [33].

### Detection of Staphylococcal Virulence and Antimicrobial Resistance Genes

Isolates were tested using conventional PCR for the presence of eleven genes (*speta*, *se-int*, *siet*, *AdsA*, *coa*, *Luk-S*, *Luk-F*, *nanB*, *spsQ*, *FnbB*, and sbi/*spsK*) coding for staphylococcus enterotoxins and proteins crucial for immune evasion and bacterial pathogenesis. The primers used for these PCRs are shown in Table 2.

**Table 2:** Primers used for the detection of virulence genes of *Staphylococcus pseudintermedius*

Gene	Primer type	Sequence (5′–3′)	Amplicon size (bp)	References
*AdsA*	Forward	GAT GCA GCC GAA CAA ACA TC	398	This study
	Reverse	ACC ATG CGA CCG TGA ATA TC		
*nanB*	Forward	CTC CAA GTA ACG ACG GGA TAA G	396	This study
	Reverse	GCT TTA GGG AGG CTG TAA GAA A		
*speta*	Forward	GGT TGT GTG CCT TAT TGG AAT G	476	This study
	Reverse	AAG CAG TTG CTG AGA TGA CTA A		
*sbi/spsK*	Forward	ATC CGA AGA GCA ACG TGA TG	674	This study
	Reverse	CAG GTG CAG ATG GAG TGT TT		
*fnbB*	Forward	GCA AAT CAA GGT GCT CAA GAA G	507	This study
	Reverse	GGA ATG ACT TCG AGA GGT TGT C		
*siet*	Forward	GCA TCT GGA GGC TAC TAC ATT T	361	This study
	Reverse	CGA TAG CGT CAA TCA AAC CAT TAC		
*se-int*	Forward	TGT TAC GCC ACC ATA CAT ACA G	336	This study
	Reverse	GAC CCA ACA CCA GAC CAA TTA		
*coa*	Forward	CAC CAC AAC AAT CTG TGC ATA C	358	This study
	Reverse	TCA CTT TCG CTT ACA CCA TAC A		
*spsQ*	Forward	ATCTCAACCTGCTCCTGATTAC	1200	[34]
	Reverse	GCATCTTTCGCTTTGTCCATAC		
*Luk-F*	Forward	CCTGTCTATGCCGCTAATCCA	572	[35]
	Reverse	AGGTCATGGAAGCTATCTCGA		
*Luk-S*	Forward	TGTAAGCAGCAGAAAATGGGG	503	[35]
	Reverse	GCCCGATAGGACTTCTTACAA		

Additionally, conventional PCR assays were performed to detect specific antimicrobial resistance genes associated with resistance to aminoglycoside, beta-lactam, colistin, fluoroquinolone, fosfomycin, fusidic acid, glycopeptide, macrolide, lincosamide, and streptogramin (MLS), nitroimidazole, oxazolidinone, phenicol, rifampin, sulfonamides, tetracycline, and trimethoprim [35–37]^40^. The primers used for these PCRs are shown in Table 3.

**Table 3:** Primers used for the detection of specific antimicrobial resistance genes of *Staphylococcus pseudintermedius*

Gene	Primer type	Sequence (5′–3′)	Amplicon size (bp)	References
*Sat4*	Forward	AAAGCAGGGCACCTGAAAGAT	344	This study
	Reverse	ATATTGATAAGCGCGCTGCC		
*CatA7*	Forward	AAACCGATACCTGAAAACACCA	180	This study
	Reverse	TACAGAATGATGAAGTTGCAGAGC		
*ant(6)-Ia*	Forward	ATCATGGAAGGTCGGCATCG	294	This study
	Reverse	CTATCCAGGCAGCCGGTTTT		
*Sul1*	Forward	AGGCTGGTGGTTATGCACTC	267	This study
	Reverse	CACCGAGACCAATAGCGGAA		
*Sul2*	Forward	TCCAGACGCTGCGTTCTATC	296	This study
	Reverse	GAAGCACCGGCAAATCGAAG		
*aac(6)-Ib4*	Forward	CATATCGTCGAGTGGTGGGG	264	This study
	Reverse	CTTGGTTCCCAAGCCTTTGC		
*CatB3*	Forward	CTGTTTCCGGACCGTGATGA	491	This study
	Reverse	ACGGCAAACTCGAGCCAATA		
*dfrG*	Forward	CGGAAGAGCCTTACCTGACA	277	This study
	Reverse	CCCTTTTTGGGCAAATACCTCA		

## Results

### Prevalence of CoPS and *S. pseudintermedius* Isolates in Pet Animals and Human Patients

Out of the 67 dogs sampled, 41 (61.2%) were found to have CoPS isolates tested by tube coagulase test. Among the human samples, 39 (22.4%) of the 174 swabs tested positive for CoPS Table 4. In total, 5 strains were confirmed as *S. pseudintermedius*—2 from apparently healthy dogs and 3 from humans—based on the amplification of the species-specific thermonuclease-encoding gene (*nuc*). Three of the five confirmed strains were included in the further analysis based on the quality of the nucleic acid: one from skin swab from an apparently healthy dog (hereafter referred to as *S. pseudintermedius* D8) and two from humans (hereafter referred to as *S. pseudintermedius* H10 and *S. pseudintermedius* H11) obtained from pus swabs from wounded patients.

**Table 4:** Number of total isolates of coagulase-positive staphylococci (CoPS) and *S. pseudintermedius* recovered from dogs and human

Host	Number of CoPS carriers (%)	Number of *S*.* pseudintermedius* positive samples
Dogs (*n* = 67)	41 (61.2%)	2
Human (*n* = 174)	39 (22.4%)	3

Through the analysis of their *nuc* genes, it was determined that the sequences of all three isolates are identical to the *nuc* gene of the *S. pseudintermedius* SP_11304-3A reference genome, confirming their species identity.

### Multilocus Sequencing Typing

The allelic profiles of the isolates used in this study were compared to those from the *S. pseudintermedius* MLST database. The three isolates presented novel sequence types, as shown in Table 5. Due to their genetic novelty, all isolates remained unassigned in the *S. pseudintermedius* MLST database.

**Table 5:** Multilocus sequencing typing (MLST) profile of the three *S. pseudintermedius* isolates

Sample	*ack*	*cpn60*	*fdh*	*pta*	*pura*	*Sar*	*tuf*	Sequence type (ST)
*S. pseudintermedius* D8	4	2	1	4	8	1	1	Unassigned
*S. pseudintermedius* H10	1	18	2	2	3	1	1	Unassigned
*S. pseudintermedius* H11	6	2	1	1	5	1	2	Unassigned

### Antibiotic Resistant Genes

**Fig. 1:**
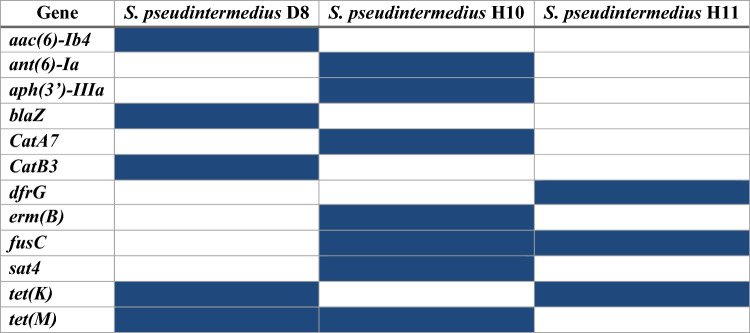
The distribution of antimicrobial resistance (AMR) genes among the isolates by real-time PCR. Genes encoding the production of beta-lactamase (*blaZ*); resistance against aminoglycosides (*aac(6)-Ib4*, *aph(3′)-III* and *ant(6′)-Ia*); chloramphenicols (*catB3 and catA7*); trimethoprim (*drfG*); macrolides (*ermB*); fusidic acid (*fusc*); streptothricin (*sat4*); and tetracyclines (*tetK*, and *tetM)*. Cells highlighted in blue indicate the presence of the corresponding gene, while white cells indicate its absence. Given that all isolates possess more than three genes associated with antimicrobial resistance, this indicates that all isolates can be classified as multidrug-resistant *Staphylococcus pseudintermedius* (MRSP)

The distribution of antimicrobial resistance (AMR) determinants varied substantially among the isolates. *S. pseudintermedius* acquires penicillin resistance by several different mechanisms, such as the production of beta-lactamase encoded by *blaZ*, which inactivates penicillin by hydrolysis of its beta-lactam ring, and the penicillin-binding protein PBP2a encoded by *mecA*, which confers methicillin resistance We did not find the *mecA* gene in the three isolates, but *blaZ* was detected in the canine isolate. The *tetK, tetL,* and *tetM* genes confer broad-spectrum resistance to tetracyclines. The *tetK* gene was detected in *S. pseudintermedius* D8 and *S. pseudintermedius* H11, while *tetM* was found in *S. pseudintermedius* D8 and *S. pseudintermedius* H10. We also found resistance genes for other antimicrobial groups. Genes that encode resistance against aminoglycosides (*aac(6)-Ib4* in *S. pseudintermedius* D8 and *aph(3′)-III* and *ant(6′)-Ia* in *S. pseudintermedius* H10), chloramphenicols (*catB3* in *S. pseudintermedius* D8 *and catA7* in *S. pseudintermedius* H10), fusidic acid (*fusc* in the two human isolates), macrolides (*ermB* in *S. pseudintermedius* H10), streptothricin (*sat4* in *S. pseudintermedius* H10), and trimethoprim (*dfrG* in *S. pseudintermedius* H11) were detected (Fig. 1).

### Virulence Genes

**Fig. 2:**
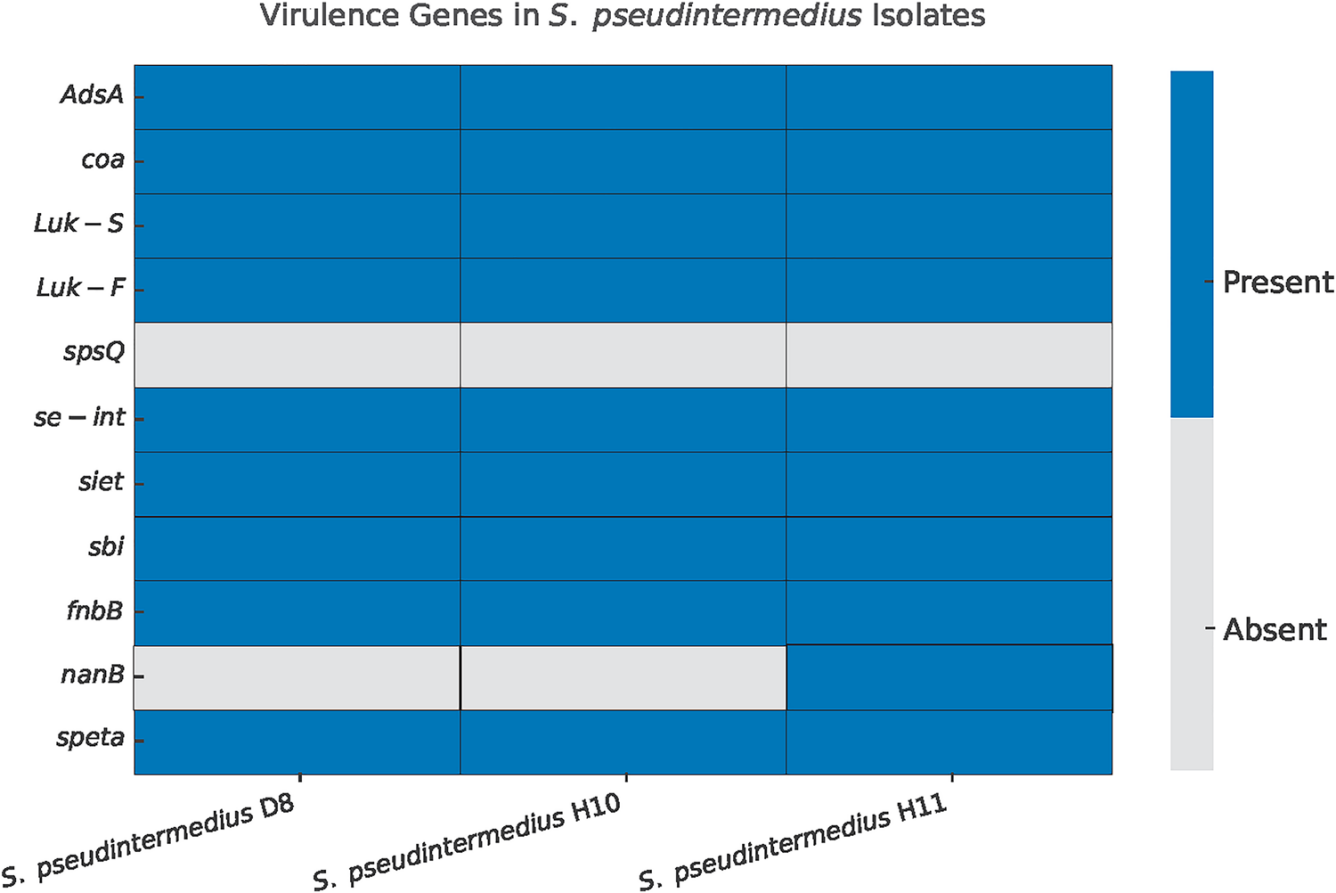
The distribution of virulence genes among the isolates determined using conventional PCR. Genes encoding the production of immune evasion (*adsA*), coagulase (*coa*), two-component pore-forming leukocidin genes (*lukF* and *lukS)*, immunoglobulin G binding protein (*spsQ*), enterotoxins (*se-int* and *siet*), fibrinogen binding protein (*fnbB*), immunoglobulin-binding protein (*sbi/spsK*), neuraminidase (*nanB*), and exfoliative toxin (*speta*) were investigated. *S. pseudintermedius* D8 is a dog sample and *S. pseudintermedius* H10 and H11 are human samples

All the isolates were positive for immune evasion (*adsA*), coagulase (*coa*), immunoglobulin-binding protein (sbi/spsK), exfoliative toxin (*speta*), enterotoxins (*se-int* and *siet*), the fibrinogen binding protein gene (*fnbB*), and two-component pore-forming leukocidin genes, *lukF* and *lukS*, were existing in all genomes. The neuraminidase gene (*nanB*) was only detected in the *S. pseudintermedius* H11 isolate and none of the isolates harbored the *spsQ*, the gene coding for the protein A, immunoglobulin G binding protein (Fig. 2).

## Discussion

*Staphylococcus pseudintermedius* is an opportunistic pathogen primarily associated with canine hosts but increasingly recognized as a zoonotic pathogen. Understanding its genetic and phenotypic characteristics, including virulence factors and antimicrobial resistance profiles, is crucial for infection control and guiding therapeutic strategies. This study provides a detailed molecular characterization of three *S. pseudintermedius* isolates from Egypt.

This study, to the best of our knowledge, is the first investigation into *S. pseudintermedius* from both human and canine hosts in Egypt, highlighting the importance of understanding zoonotic transmission and its implications for pet owners and veterinary staff. MLST analysis revealed that all three isolates presented novel sequence types (STs), remaining unassigned in the *S. pseudintermedius* MLST database. An"unassigned" ST indicates that these isolates have unique allelic profiles not previously identified.

A recent study in Spain also identified diverse MLST profiles among *S. pseudintermedius* isolates, discovering several novel STs [38]. Similarly, research in the United States has shown high population diversity of *S. pseudintermedius* in companion animals, with evidence of clonal expansion of some MRSP lineages inside each area [39]. A study of 155 *S. pseudintermedius* isolates from companion animals in Lisbon, Portugal, found that 31.0% were methicillin-resistant (MRSP), of which 95.8% exhibited multidrug-resistant (MDR) phenotypes; using MLST, the study identified 42 clonal lineages, including 25 new sequence types (STs) [40]. Additionally, a comprehensive review of *S. pseudintermedius* MLST data indicated a high level of genetic diversity across different geographical regions [41]. A study conducted in South Africa showed minimal seasonal variation in the number of Staphylococcus isolates and highlighted a diverse range of Staphylococcus species present among dogs [42]. Taken together these findings emphasize the importance of continuous surveillance and highlight the need for accurate diagnosis and proper therapy selection.

The *mecA* gene, responsible for methicillin resistance, was absent in all three isolates, which contrasts with findings from recent studies in the United States and other regions where *mecA* is commonly detected [29, 43, 44]. The detection of the *bla*Z gene in the canine isolate aligns with studies from Europe and parts of Africa, where beta-lactamase-mediated penicillin resistance is prevalent [45, 46].

The presence of *tet*K and *tet*M genes, conferring tetracycline resistance, is consistent with reports from Africa and Scotland, indicating a global spread of these resistance determinants [45, 47]. Furthermore, the identification of resistance genes for aminoglycosides, chloramphenicol, fusidic acid, macrolides, and streptothricin reflects a multifaceted AMR profile similar to those observed in diverse geographical regions [45, 47]. Given that all isolates possess more than three genes associated with antimicrobial resistance, this indicates that all isolates can be classified as multidrug-resistant *Staphylococcus pseudintermedius* (MRSP). These findings underscore the necessity of regional antibiotic stewardship policies to effectively manage the spread of resistant strains.

The isolates were positive for several key virulence genes, including *ads*A, *coa*, *sbi*/*sps*K, *spet*a, *se-int* and* siet*, *fnb*B, and *luk*F and *luk*S. These genes play crucial roles in the pathogenicity of *S. pseudintermedius*, facilitating immune evasion, tissue adhesion, and toxin-mediated damage [48].

Interestingly, none of the isolates harbored *sps*Q, the gene coding for immunoglobulin G binding protein (protein A). Protein A is known for its role in immune evasion by binding to the Fc region of antibodies, inhibiting opsonization and phagocytosis [48]. The lack of *sps*Q might imply alternative immune evasion strategies employed by these isolates, warranting further investigation.

The current study has some limitations, including the relatively small sample size and the narrow geographic scope (one governorate); therefore, we plan to perform a larger study that includes more samples of a variety of types from multiple Egyptian governorates. This will provide a more comprehensive picture of the epidemiology of *S. pseudintermedius* infections in Egypt. In addition, the isolation protocol did not include a preliminary selective enrichment step to favor staphylococcal growth. This approach was chosen to harmonize specimen processing with the protocol routinely applied by the local laboratory, which does not employ selective enrichment. However, we acknowledge that the absence of such an enrichment step represents a limitation of the present study, as environmental bacteria may overgrow staphylococci.

## Conclusion

This study focuses on the identification and detailed molecular characterization of three *S. pseudintermedius* isolates from both canine and human sources in Egypt. Given the close interaction between dogs and humans, the zoonotic potential of *S. pseudintermedius* poses significant public health concerns, particularly for pet owners and veterinary staff. Understanding the genetic and phenotypic properties of this pathogen is crucial for developing effective infection control strategies and guiding therapeutic interventions.

## Data Availability

The datasets generated during and/or analyzed during the current study are available from the corresponding author on reasonable request.
